# Toxicokinetics under extracorporeal clearance and temporal dissociation of hepatotoxicity and coagulopathy in ACDMB poisoning: Two index cases

**DOI:** 10.1016/j.toxrep.2026.102277

**Published:** 2026-05-19

**Authors:** Kefeng Jin, Peng Wang, Xiuqin Yang, Xiaofei Zhang, Tao Huang, Yue Qiu, Mo Li, Yuan Wang, Xue Ma

**Affiliations:** a Department of Nephrology, Shizuishan First People's Hospital, Shizuishan, Ningxia 753200, China; b Zhejiang Provincial Center for Disease Control and Prevention, Hangzhou, Zhejiang 310051, China

**Keywords:** ACDMB, Toxicokinetics, Coagulation reserve index, Hemoperfusion, Acute liver failure

## Abstract

**Background:**

2-Amino-5-chloro-N,3-dimethylbenzamide (ACDMB), a synthetic intermediate for the insecticide chlorantraniliprole, causes extremely rare occupational poisoning (six cases worldwide) with unknown human toxicokinetics. We report two consecutive index cases from the same manufacturing facility.

Case presentation

Case A (22-year-old male) and Case B (37-year-old male) developed fulminant hepatitis following brief accidental occupational exposure. Both received plasma exchange and continuous venovenous hemofiltration; Case A additionally received hemoperfusion. Both achieved full recovery and were discharged on Day 28 (Case A) and Day 14 (Case B), respectively.

**Results:**

Serial serum monitoring in Case B revealed mono-exponential elimination (apparent half-life 18.4 h, 95% CI 15.3–22.1). Both patients exhibited temporal dissociation: peak hepatotoxicity occurred at 54–65 h, whereas critical coagulopathy (Coagulation Reserve Index [CRI] <5) developed later at 123–127 h when > 95% of toxin had been cleared. At comparable CRI nadirs, hemoperfusion in Case A resulted in immediate circuit clotting and prolonged DIC reversal (48 h), whereas cryoprecipitate support in Case B achieved rapid recovery (8.3 h) without adsorptive therapy.

**Conclusion:**

These observations provide the first human ACDMB toxicokinetic profile under extracorporeal clearance and generate the hypothesis that CRI < 5 may identify high-risk periods for hemoperfusion, though prospective validation is required.

## Highlights

• First human ACDMB toxicokinetics: initial t½ ∼9–10 h, extracorporeal t½ 18.4 h.
• Coagulation Reserve Index (CRI) < 5 indicates high-risk period for hemoperfusion.
• Temporal dissociation (∼60 h) between hepatotoxicity and coagulopathy.
• Hemoperfusion failure vs. cryoprecipitate success when CRI < 5.
• Cases 5 and 6 of only six documented ACDMB poisonings worldwide.

## Graphical Abstract

**Figure:**
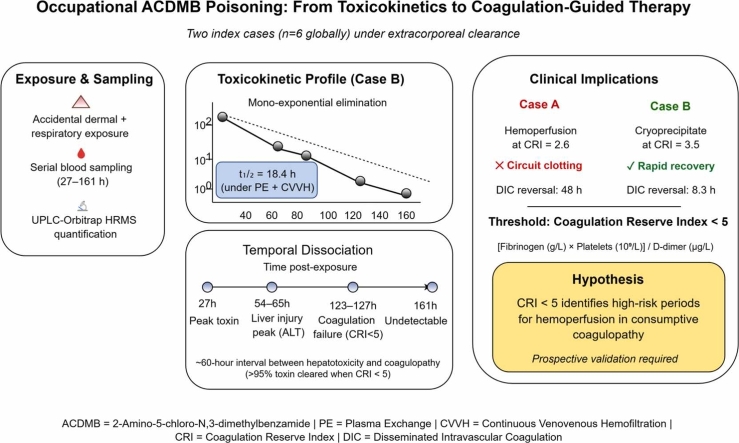

## 1 Introduction

Amino-5-chloro-N,3-dimethylbenzamide (ACDMB), a key intermediate in the synthesis of the insecticide chlorantraniliprole, has emerged as a novel occupational hepatotoxin with unknown human toxicokinetics [1]. Industrial synthesis occurs under strictly controlled closed-system conditions, and the compound has no commercial or household applications, accounting for the extreme rarity of human exposure (only six documented cases globally, including the two reported here) [1]. Preclinical toxicology data remain limited; available safety data indicate low acute oral toxicity (LD₅₀ > 2000 mg/kg in rats) and no mutagenic potential in standard Ames testing, yet no peer-reviewed inhalation or repeated-dose studies have been published [1], [2]. Feng et al. recently reported the first clinical series of four patients (Cases 1–4 globally) who developed fulminant hepatitis following accidental occupational exposure, establishing the clinical pentad of latency, rash, fever, liver injury, and recovery, with histopathological evidence of mitophagy as a potential mechanism [3]. The clinical presentation resembles other causes of acute liver failure [4], yet with distinctive temporal dissociation between hepatic and coagulation failure. However, critical knowledge gaps persist: dynamic toxin clearance profiles remain undefined, as blood concentrations were measured only at single timepoints upon admission; the temporal relationship between hepatotoxicity and coagulopathy was uncharacterized; and pharmacokinetic parameters such as elimination half-life during extracorporeal therapy are entirely unknown.

We herein report Cases A and B globally, comprising 33% of the cumulative experience with ACDMB poisoning. Unlike the initial four cases reported by Feng et al. [3] in which toxin concentrations were measured only at single timepoints upon admission,Case B underwent serial high-resolution monitoring (5 timepoints, 27–161 h) using UPLC-Q-Orbitrap HRMS [5], providing the first human elimination half-life data. We also introduce the Coagulation Reserve Index (CRI = [fibrinogen (g/L) × platelets (10⁹/L)] / D-dimer [µg/L]) as an exploratory bedside metric to describe coagulation trajectories and generate hypotheses regarding hemoperfusion safety during consumptive coagulopathy.

This investigation represents a descriptive case series of an ultra-rare poisoning (cumulative global incidence n = 6). We explicitly acknowledge that observations derived from two index cases cannot establish causality and require prospective validation in larger cohorts before clinical implementation [6]^.^

### 1.1 Case presentation

Both cases occurred at the same chemical manufacturing facility producing chlorantraniliprole intermediates in Shizuishan, Ningxia, China. Case A (December 2024) involved a 22-year-old male worker who accidentally entered the production area without protective equipment for approximately 2–3 min, sustaining both respiratory exposure and a 5 cm × 4 cm chemical burn on the neck. Case B (May 2025), a 37-year-old male coworker, was exposed for approximately

2 min after removing his respirator inside the production unit. Both incidents were accidental breaches of standard occupational safety protocols. The facility had experienced no previously documented ACDMB intoxications, although environmental screening conducted after these incidents revealed persistent low-level workplace contamination. Both patients presented with

irritant symptoms including nausea, vomiting, fever, and rash; however, no severe respiratory mucosal injury (e.g., chemical pneumonitis) was observed beyond the neck dermal burn in Case A.

#### 1.1.1 Case A

A 22-year-old male manufacturing worker presented 53 h after occupational dermal and respiratory exposure to ACDMB. On admission, he developed fever, rash, and rapidly progressive jaundice. Laboratory studies revealed fulminant hepatic injury (peak alanine aminotransferase [ALT] 3553 U/L at 54 h) and overt disseminated intravascular coagulation (DIC; ISTH-DIC score ≥5) [7]. The Coagulation Reserve Index (CRI), calculated as [fibrinogen (g/L) × platelets (10⁹/L)] / D-dimer (µg/L), was 14.9 at 54 h post-exposure.

The patient received plasma exchange (PE; 2 L plasma volume per session) and continuous venovenous hemofiltration (CVVH; 35 mL/kg/h). Due to persistent thrombocytopenia, three sessions of hemoperfusion (HP; HA330 resin cartridges) were added. During the first HP session (60–68 h), CRI remained > 14. However, by the second HP session (85–89 h), CRI had declined to 5.3, coinciding with worsening thrombocytopenia (platelets 45 ×10⁹/L at 71 h). Recombinant human thrombopoietin (15,000 U/d) was administered from 71 to 123 h [8], achieving transient platelet recovery (63 ×10⁹/L at 127 h).

At 127 h post-exposure, with CRI critically depleted to 2.56 and platelets at 43 × 10⁹/L, a third HP session was initiated. Within 1 h, massive circuit clotting occurred despite nafamostat anticoagulation, necessitating immediate termination. Notably, preceding plasma exchange sessions had been completed successfully; the circuit failure was specific to hemoperfusion under critical coagulopathy. No further HP was attempted.

Following permanent discontinuation of hemoperfusion at 127 h, the patient received continued plasma exchange and CVVH without further adsorptive therapy. Under recombinant human thrombopoietin support (71–123 h), platelets recovered progressively from 36 × 10⁹/L (123 h) to 63 × 10⁹/L (148 h), 73 × 10⁹/L (176 h), and 104 × 10⁹/L (217 h). A complete coagulation panel at 217 h confirmed substantial recovery (fibrinogen 2.06 g/L, platelets 204 ×10⁹/L, D-dimer 1.53 mg/L FEU, PT 10.8 s, INR 1.00), indicating restored coagulation reserve. Alanine aminotransferase declined from 2491 U/L (123 h) to 251 U/L (148 h), 86 U/L (176 h), and 36 U/L (217 h), with full normalization to 42 U/L by discharge on Day 28.

#### 1.1.2 Case B

A 37-year-old male coworker (similar exposure route) presented at 27 h post-exposure. Serum ACDMB concentration was 346.1 µg/L (peak), and CRI was 118. He developed comparable hepatic injury (peak ALT 5871 U/L at 65 h) and overt DIC (ISTH-DIC score 7).

This patient received identical PE and CVVH therapy but did not receive hemoperfusion. Following PE initiation at 47 h, serum ACDMB concentrations declined rapidly: 19.5 µg/L at 65 h (94% reduction) and 13.1 µg/L at 88 h (96% reduction). CRI declined from 118 to 33 by 65 h, then transiently improved to 91 at 88 h following PE and CVVH.

By 114 h, CRI had fallen to 13.4, and by 127 h, it reached 3.5 (comparable to Case A’s nadir of 2.56). At this critical juncture, instead of hemoperfusion, the clinical team administered cryoprecipitate (10 units) and fresh frozen plasma [9]. This proactive coagulation support, unaccompanied by adsorptive therapy, was followed by rapid CRI recovery to 8.5 by 135 h. DIC resolved within 8.3 h—substantially faster than Case A. Serum ACDMB became undetectable (<0.36 µg/L) by 161 h.

### 1.2 Comparative summary

Both patients exhibited similar temporal patterns: ALT peaked at 54–65 h, while critical coagulopathy (CRI <5) manifested later (>120 h) when > 95% of toxin had been cleared. However, at the critical 127 h timepoint, divergent management yielded contrasting outcomes: hemoperfusion in Case A resulted in immediate circuit failure and prolonged DIC (48 h), whereas cryoprecipitate support in Case B achieved rapid recovery (8.3 h) without adsorptive therapy.

### 1.3 Clinical outcome

Both patients achieved complete recovery. Case A was discharged on Day 28 with normalized liver enzymes (ALT 42 U/L) and coagulation parameters (platelets 198 ×10⁹/L, fibrinogen 3.5 g/L). Case B was discharged on Day 14 with ALT 220 U/L and fully restored coagulation function; ALT normalized to 46 U/L at 1-week outpatient review. At 3-month follow-up, neither patient exhibited residual hepatic dysfunction, coagulopathy, or other sequelae. Both have returned to their previous occupations (Case B with strict respiratory protection; Case A transferred to a non-exposure position).

## 2 Methods

### 2.1 Study design and ethics

This descriptive case series was approved by the Institutional Review Board of Shizuishan First People's Hospital (Approval No. 2025–0601). Written permission for data use was obtained from both patients.

### 2.2 Sample collection and storage

Serum samples for ACDMB analysis were obtained from clinical remnant specimens (excess material from routine biochemical monitoring) collected during the acute phase of illness. Samples were stored at −20°C in the clinical laboratory during active treatment, then transferred to −80°C upon initiation of the research protocol for long-term preservation. Sampling timepoints were verified against clinical records (admission, transfer, and monitoring timestamps). While these represent opportunistic use of clinical waste material rather than prospectively collected research samples, the documented collection times and strict cold-chain maintenance ensure data integrity. ACDMB concentrations were not available to clinicians during treatment decision-making; therapeutic interventions were based solely on standard clinical parameters (ALT, coagulation profiles).

### 2.3 Toxin analysis

Serum ACDMB concentrations were quantified by UPLC-Q-Orbitrap HRMS [5]. Briefly, 200 µL serum was precipitated with 800 µL acetonitrile, centrifuged (3500 rpm, 15 min), and analyzed using targeted MS² mode (precursor *m/z* 199.06371; product *m/z* 168.02144). The lower limit of quantification was 0.36 µg/L (intra-day CV <3.0%, inter-day CV <3.5%). Serial serum ACDMB quantification was performed only in Case B, as these measurements relied on retrospectively collected clinical remnant specimens. Case B presented at 27 h post-exposure, allowing serial sampling from peak concentration through elimination; Case A presented at 53 h, after the early phases had elapsed, and no corresponding remnant specimens were available. Thus, Case B provided the toxicokinetic profile, while Case A served as the clinical comparator for coagulation trajectories. Serial sampling was performed at 27, 65, 88, 127, and 161 h post-exposure in Case B.

### 2.4 Blood purification

Both patients received plasma exchange (2 L plasma volume per session) and continuous venovenous hemofiltration (35 mL/kg/h, AV600S polysulfone filters) via central venous access with nafamostat anticoagulation [10]. Case A additionally received hemoperfusion (HA330 resin cartridges [11]) during three sessions. Detailed schedules are provided in Supplementary Table 1.

### 2.5 Coagulation reserve index

CRI was calculated retrospectively as [fibrinogen (g/L) × platelets (10⁹/L)] / D-dimer (µg/L). This exploratory metric integrates consumption and fibrinolysis; CRI < 5 was observed coinciding with urgent hemostatic support. The CRI incorporates fibrinogen as a key marker of consumptive coagulopathy [12], reflecting the critical role of fibrinogen depletion in DIC severity assessment.

### 2.6 Toxicokinetic analysis

**Fig. 1:**
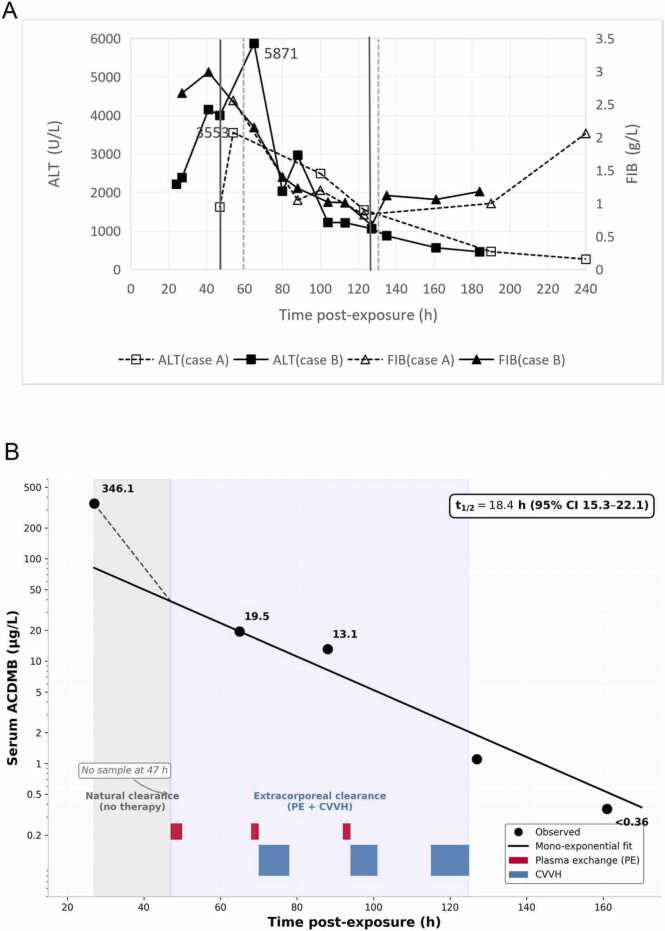
**A.** Temporal dissociation between hepatotoxicity and coagulopathy in two patients with occupational ACDMB poisoning. Left Y-axis: alanine aminotransferase (ALT, U/L) for Case B (solid line with solid squares) and Case A (dashed line with open squares). Right Y-axis: fibrinogen (FIB, g/L) for Case B (solid line with solid triangles) and Case A (dashed line with open triangles). The interval between the two vertical solid lines denotes the extracorporeal clearance period in Case B (PE + CVVH, 47–125 h); the interval between the two vertical dashed lines denotes the extracorporeal clearance period in Case A (PE + CVVH, 60–128 h). Both patients exhibited a consistent temporal dissociation: peak hepatotoxicity occurred at 54 h (ALT 3553 U/L, Case A) and 65 h (ALT 5871 U/L, Case B), whereas fibrinogen nadirs and overt coagulopathy developed later (>120 h).Fig. 1**B.** Serum ACDMB concentration–time profile in Case 2. The shaded area (27–47 h) denotes the uninfluenced natural clearance phase prior to extracorporeal therapy; the lightly shaded area (47–125 h) indicates the extracorporeal clearance phase. The mono-exponential elimination curve (solid line; apparent t½ 18.4 h, 95% CI 15.3–22.1) was fitted to concentrations during the post-treatment observation window (47–161 h). Dashed line indicates projected trend during 27–47 h without a confirmed 47-h measurement. Red bars, plasma exchange (PE: 47–50, 68–70, and 92–94 h); blue bars, continuous venovenous hemofiltration (CVVH: 70–78, 94–101, and 115–125 h). The concentration at 88 h transiently exceeds the fitted curve, likely reflecting residual extracorporeal clearance from the preceding CVVH session (70–78 h).

A mono-exponential elimination model was fitted to serial concentrations from Case B. Toxicokinetic analysis was stratified into two distinct phases: (i) the uninfluenced natural clearance phase (27--47 h post-exposure), during which no extracorporeal therapy was administered; and (ii) the extracorporeal clearance phase (47--161 h), encompassing intermittent plasma exchange and CVVH. The apparent elimination half-life (t½ 18.4 h, 95% CI 15.3--22.1) was derived from mono-exponential fitting during the extracorporeal phase (65--161 h). Projecting the fitted mono-exponential trend backward to the uninfluenced natural clearance interval (27--47 h; dashed line in Fig. 1B) yields an estimated intrinsic half-life of approximately 8--9 h, consistent with more rapid pre-treatment elimination prior to plasma exchange initiation.

## 3 Results

### 3.1 Toxicokinetics (Case B)

Serum ACDMB concentration exhibited smooth mono-exponential elimination (R² > 0.95; Fig. 1B, Supplementary Table 2), declining from a peak of 346.1 µg/L at 27 h post-exposure (prior to extracorporeal therapy initiation) to < 0.36 µg/L by 161 h. The consistency of this elimination curve and its temporal correlation with clinical markers (ALT peak at 65 h) support the reliability of retrospectively analyzed samples. Following commencement of plasma exchange and continuous venovenous hemofiltration at 47 h, concentration declined to 19.5 µg/L by 65 h (94.4% reduction) and further to 13.1 µg/L by 88 h (96.2% clearance); by 127 h, only 1.1 µg/L remained. Non-linear regression analysis revealed an apparent elimination half-life (t½) of 18.4 h (95% CI 15.3–22.1) during the extracorporeal clearance phase (47–161 h).

### 3.2 Coagulation reserve index trajectories

**Fig. 2:**
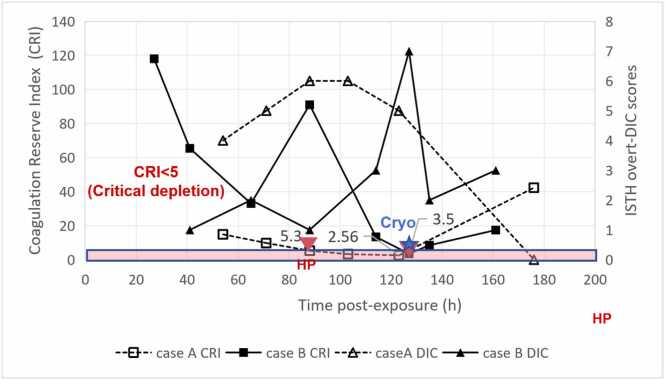
Concordance between Coagulation Reserve Index (CRI) and ISTH overt-DIC scores in two patients receiving plasma exchange and continuous venovenous hemofiltration (CVVH). Left Y-axis: CRI for Case B (solid line with solid squares) and Case A (dashed line with open squares). Right Y-axis: ISTH overt-DIC scores for Case B (solid line with solid triangles) and Case A (dashed line with open triangles). The red shaded area indicates the critical depletion zone (CRI < 5). Red inverted triangles (HP) indicate hemoperfusion sessions in Case A at CRI 5.3 and 2.56; blue star (Cryo) indicates cryoprecipitate administration in Case B at CRI 3.5. Note the inverse relationship between CRI and DIC trajectories during critical depletion, generating hypotheses regarding intervention timing, though unmeasured confounders preclude causal inference.

Both patients exhibited dynamic CRI trajectories with critical divergence at comparable nadir values (Supplementary Table 2**,** Fig. 2). In Case A, CRI declined progressively from 14.5 at 48 h to 5.3 at 88 h (during the second hemoperfusion session), reaching 2.56 at 123 h. At 127 h, when hemoperfusion was initiated at CRI 2.56, massive circuit clotting occurred within 1 h, necessitating immediate termination. in Case B, CRI declined from 118 at 27 h to 33 at 65 h, with transient recovery to 91 at 88 h following plasma exchange. CRI subsequently fell to 13.4 at 114 h and reached 3.5 at 127 h—comparable to Case A’s nadir. At this critical timepoint, cryoprecipitate administration (10 units) was followed by recovery to 8.5 by 135 h and 42.3 by 176 h.

### 3.3 Temporal dissociation of hepatotoxicity and coagulopathy

Peak hepatotoxicity (ALT 5871 U/L in Case B at 65 h; 3553 U/L in Case A at 54 h) preceded critical coagulopathy (CRI <5) by approximately 60 h(Fig. 1**A and** Fig. 2). In both patients, CRI fell below 5 when > 95% of toxin had been eliminated (Case 2: <1.1 µg/L at 127 h; Case A: presumably cleared at 123 h). This consistent interval suggests dissociation between direct toxic effects and secondary consumptive coagulopathy.

### 3.4 Comparative outcomes

Despite comparable CRI nadirs (<5) at 127 h, divergent management yielded contrasting recovery trajectories(Fig. 2). Case A (hemoperfusion at CRI 2.56) exhibited prolonged DIC reversal (48 h) and nadir platelets of 36 × 10⁹/L.Case B (cryoprecipitate support at CRI 3.5, no hemoperfusion) achieved faster DIC resolution (8.3 h) with nadir platelets of 43 × 10⁹/L.

## 4 Discussion

### 4.1 Principal findings and knowledge contributions

This report provides three essential additions to the literature on ACDMB poisoning, which remains extremely rare (cumulatively 6 cases worldwide). First, we establish the first human toxicokinetic profile of ACDMB, demonstrating mono-exponential elimination with a approximative half-life of 18.4 h (95% CI 15.3–22.1)—substantially longer than conventional hepatotoxins such as acetaminophen (t½ 2–4 h) [13], [14].Case B achieved complete toxin clearance (>95% by 88 h, undetectable by 161 h) without hemoperfusion, suggesting that plasma exchange-based therapy may be sufficient for this poisoning [15], [16]. Second, we provide proof-of-concept that serial UPLC-Q-Orbitrap monitoring is feasible in acute occupational poisoning, enabling precise pharmacokinetic analysis. Third, we introduce preliminary evidence that coagulation reserve trajectories may guide extracorporeal therapy timing.

Both patients exhibited a consistent ∼60-hour interval between peak hepatotoxicity (ALT 54–65 h) and critical coagulopathy onset (>120 h), occurring when > 95% of toxin had been eliminated. This temporal dissociation suggests a secondary consumptive process independent of direct toxicodynamic effects [17]. The Coagulation Reserve Index (CRI) tracked this trajectory: in Case A, CRI declined to 2.56 at 127 h, coinciding with massive circuit clotting within 1 h of hemoperfusion initiation despite anticoagulation. In contrast,Case B (managed without hemoperfusion) received cryoprecipitate support at comparable CRI depletion (3.5), achieving faster DIC reversal (8.3 h vs. 48 h). These observations generate the hypothesis that CRI < 5 may identify periods when hemoperfusion becomes technically unfeasible due to consumptive coagulopathy.

### 4.2 Clinical implications

The contrasting trajectories observed when CRI fell below 5 generate the hypothesis that hemoperfusion may exacerbate consumptive coagulopathy in critically depleted states. The integration of consumption markers (fibrinogen, platelets) and fibrinolysis (D-dimer) into CRI reflects the pathophysiology of inflammation-induced coagulopathy [18], while the trajectory from intact reserve to critical depletion (<5) mirrors the sepsis-induced coagulopathy to overt DIC continuum [19]. Resin-based hemoperfusion cartridges adsorb fibrinogen and platelets alongside toxins [20]; when superimposed on pre-existing DIC at CRI < 5, this creates additive hemostatic stress that may outweigh the incremental benefit of adsorptive therapy once PE+CVVH achieves adequate toxin clearance (>95%) [21].

Unlike static DIC scores, this calculable framework ([fibrinogen × platelets]/D-dimer) offers bedside risk stratification to identify optimal windows for invasive extracorporeal therapy. We emphasize that CRI remains an unvalidated exploratory metric; the proposed threshold of < 5 derives from post-hoc observation in these two index cases alone and requires prospective derivation in broader cohorts before clinical implementation.

### 4.3 Limitations

This hypothesis-generating study of two index cases cannot establish causality; the contrasting trajectories reflect unmeasured confounders (potential differences in exposure dose, time-to-treatment, and endogenous clearance capacity) inherent to observational rare disease research. Second, the CRI requires validation in independent DIC cohorts; we explicitly discourage extrapolation to other poisonings or patient populations without prospective testing. Third, pharmacokinetic parameters were derived from one patient with complete sampling; variability in protein binding or renal function may alter elimination kinetics in other cases. Fourth, ACDMB concentrations were measured from retrospectively collected clinical remnant specimens rather than prospectively acquired research samples. While strict cold-chain maintenance (-20°C to −80°C) preserves analyte stability, these data were unavailable during clinical decision-making, limiting our ability to assess whether real-time toxicokinetic monitoring would have altered therapeutic strategy. These constraints position our findings as foundational observations requiring validation, rather than guidelines for clinical practice.

## CRediT authorship contribution statement

**Xiuqin Yang:** Supervision, Resources, Investigation, Data curation. **Xiaofei Zhang:** Resources, Investigation. **Tao Huang:** Resources, Investigation, Data curation. **Yue Qiu:** Resources, Investigation. **Kefeng Jin:** Writing – review & editing, Writing – original draft, Supervision, Resources, Project administration, Methodology, Investigation, Conceptualization. **Peng Wang:** Writing – review & editing, Methodology, Investigation, Formal analysis, Data curation. **Mo Li:** Resources, Investigation. **Yuan Wang:** Resources, Methodology, Investigation. **Xue Ma:** Resources, Investigation.

## Declarations ethics approval and consent to participate

This study was approved by the Institutional Review Board of Shizuishan First People's Hospital (Approval No. 2025–0601). Written informed consent for publication of de-identified clinical data and images was obtained from both patients. A copy of the consent form is available for review by the Editor-in-Chief upon request.

## Clinical Trial Registration

This study was registered with the Chinese Clinical Trial Registry (ChiCTR230000290152).

## Consent for Publication

Written informed consent was obtained from the patients for publication of this case report and accompanying data/images.

## Funding

This research received no specific grant from any funding agency in the public, commercial, or not-for-profit sectors. The UPLC-Q-Orbitrap HRMS analytical platform was supported by the Zhejiang Province Medical and Health Science and Technology Plan (Grant Nos. 2022KY132, 2024KY903) for method development and validation.

## Declaration of Competing Interest

The authors declare the following financial interests/personal relationships which may be considered as potential competing interests: Kefeng Jin has patent Coagulation Reserve Index (CRI)-guided therapeutic strategy pending to Patent application CN 2025114441440. If there are other authors, they declare that they have no known competing financial interests or personal relationships that could have appeared to influence the work reported in this paper.

## Data Availability

Data will be made available on request.

## Footnotes

Appendix A Supplementary data associated with this article can be found in the online version at doi:10.1016/j.toxrep.2026.102277.
