# Supplementary material for: Toxicokinetics under extracorporeal clearance and temporal dissociation of hepatotoxicity and coagulopathy in ACDMB poisoning: Two index cases
Source: Toxicol Rep. 2026 May 19;16:102277. doi: 10.1016/j.toxrep.2026.102277 (PMC13224010; doi:10.1016/j.toxrep.2026.102277)
Supplement: Supplementary file 1 — Supplementary material [file mmc1.docx]

**Supplementary Table 1. Chronological Record of Blood Purification Procedures and Clinical Trajectories**

| Time post-exposure (h) | Case A (PE+CVVH+HP) | Case B (PE+CVVH) | Phase/Critical event |
| --- | --- | --- | --- |
| T47-50h | - | PE 3h | Initiation of extracorporeal therapy |
| T60-63h | PE 3h | - | - |
| T63-68h | HP+CVVH 4.5h | - | First HP session (Case 1) |
| T68-70h | - | PE 2h |  |
| T71 | TPO initiated (15,000 U/d)¥ |  | Platelets 45×10⁹/L (Case 1) |
| T70-78h | - | CVVH 8h | CRI: 33 → 91 (Case 2) |
| T82-85h | PE 3h | - | - |
| T85-89h | HP + CVVH 4h | - | CRI decline: 9.8 → 5.3 (Case 1) |
| T87-88h | HP in progress | Serum ACDMB 13.1 µg/L | - |
| T92-94h | - | PE 2h | - |
| T94-101h | - | CVVH 7h | CRI: 91 → 13.4 (Case 2) |
| T115-125h | - | CVVH 9.5h | CRI: 13.4 → 3.5 (Case 2) |
| T127h | TPO discontinued (platelets 63) |  | Platelet recovery peak (Case 1) |
| T124-127h | PE 3h | - | - |
| T127-128h | HP 1 h (terminated)**⚡** | CVVH ongoing | Coagulopathy peak (both cases) |
| T127h | CRI 2.56 | CRI 3.5; Cryoprecipitate | Critical intervention divergence |
| T128h | Termination of HP | Continued CVVH | CRI recovery: 3.5 → 17.9 (Case 2) |
| T161h | - | Serum ACDMB <0.36 µg/L | End of observation |

**Abbreviations:** CVVH, continuous venovenous hemofiltration; h, hours; HP, hemoperfusion; PE, plasma exchange.

**Note:** CRI, Coagulation Reserve Index (calculated as [fibrinogen (g/L) × platelets (10⁹/L)] / D-dimer (µg/L)); arrow (→) indicates trend during the time interval.

¥ TPO: thrombopoietin, 15,000 U daily × 3 days (T71-T123). ⚡ HP terminated due to circuit clotting despite nafamostat anticoagulation.

**⚡** HP terminated due to circuit clotting despite nafamostat anticoagulation.

**Supplementary Table 2. Key Timepoints of Toxicokinetics, Coagulation Reserve, and Interventions**

| Time post-exposure**¶** (h) | Serum ACDMB (µg/L) | Case A (PE+CVVH+HP) | | Case B (PE+CVVH) | |
| --- | --- | --- | --- | --- | --- |
|  | - | CRI | Key intervention | CRI | Key intervention |
| 27 | 346.1(Peak) | - | - | 118 | Admission (PE initiated at T47) |
| 54 | - | 14.9 | Pre-treatment (PE+HP pending) | - | - |
| 65 | 19.5† | - | - | 33 | PE+CVVH ongoing |
| 88 | 13.1 ‡ | 5.3 | HP in progress (2nd session) | 91 | Inter-treatment interval |
| 127 | 1.1 | **2.56**** | HP terminated (3rd, 1h only, due to circuit clotting) | **3.5**** | Cryoprecipitate 10 U |
| 161 | <0.36 (LOQ) | - | - | 17.9 | Recovery phase |

**Abbreviations:** ACDMB, 2-amino-5-chloro-N,3-dimethylbenzamide; CRI, Coagulation Reserve Index; CVVH, continuous venovenous hemofiltration; HP, hemoperfusion; LOQ, limit of quantification (0.36 µg/L); PE, plasma exchange; U, units.

**Notes:**

† Denotes 94.4% clearance from peak;

‡ Denotes 96.2% clearance from peak;

¶ Time zero represents the estimated time of occupational exposure.

**— indicates no sampling or intervention at this timepoint; serum ACDMB was not measured in Case 1.**

**CRI values <5 are shown in bold, indicating critical coagulation reserve depletion.

**†† Case 1 received thrombopoietin (TPO) 15,000 U/d from T71 to T123 (platelets recovered from 45 to 63 ×10⁹/L).**
